# Medium-Chain Length Fatty Acids Enhance Aβ Degradation by Affecting Insulin-Degrading Enzyme

**DOI:** 10.3390/cells10112941

**Published:** 2021-10-29

**Authors:** Janine Mett, Anna A. Lauer, Daniel Janitschke, Lea V. Griebsch, Elena L. Theiss, Heike S. Grimm, Hennariikka Koivisto, Heikki Tanila, Tobias Hartmann, Marcus O. W. Grimm

**Affiliations:** 1 Biosciences Zoology/Physiology-Neurobiology, Faculty NT-Natural Science and Technology, Saarland University, 66123 Saarbrücken, Germany; janine.mett@uni-saarland.de; 2 Experimental Neurology, Saarland University, 66424 Homburg, Germany; anna.lauer@uks.eu (A.A.L.); daniel.janitschke@uks.eu (D.J.); lea@ifuws.de (L.V.G.); elena.theiss@web.de (E.L.T.); heike.grimm@gmx.de (H.S.G.); tobias.hartmann@uks.eu (T.H.); 3 A.I. Virtanen Institute, University of Eastern Finland, 70211 Kuopio, Finland; henna.koivisto@uef.fi (H.K.); heikki.tanila@uef.fi (H.T.); 4 Deutsches Institut für Demenzprävention, Saarland University, 66424 Homburg, Germany; 5 Nutrition Therapy and Counseling, Campus Rheinland, SRH University of Applied Health Sciences, 51377 Leverkusen, Germany

**Keywords:** Aβ degradation, insulin-degrading enzyme, Alzheimer´s disease, fatty acids, medium-chain fatty acids, lauric acid, myristic acid, coconut oil

## Abstract

The accumulation of amyloid β-protein (Aβ) is one of the major pathological hallmarks of Alzheimer’s disease. Insulin-degrading enzyme (IDE), a zinc-metalloprotease, is a key enzyme involved in Aβ degradation, which, in addition to Aβ production, is critical for Aβ homeostasis. Here, we demonstrate that saturated medium-chain fatty acids (MCFAs) increase total Aβ degradation whereas longer saturated fatty acids result in an inhibition of its degradation, an effect which could not be detected in IDE knock-down cells. Further analysis of the underlying molecular mechanism revealed that MCFAs result in an increased exosomal IDE secretion, leading to an elevated extracellular and a decreased intracellular IDE level whereas gene expression of IDE was unaffected in dependence of the chain length. Additionally, MCFAs directly elevated the enzyme activity of recombinant IDE, while longer-chain length fatty acids resulted in an inhibited IDE activity. The effect of MCFAs on IDE activity could be confirmed in mice fed with a MCFA-enriched diet, revealing an increased IDE activity in serum. Our data underline that not only polyunsaturated fatty acids such as docosahexaenoic acid (DHA), but also short-chain fatty acids, highly enriched, for example in coconut oil, might be beneficial in preventing or treating Alzheimer’s disease.

## 1. Introduction

One of the characteristic hallmarks of Alzheimer´s disease (AD) is the progressive deposition of extracellular amyloid plaques in brain regions responsible for memory and cognition such as the hippocampus and cortex. The major component of amyloid plaques is the hydrophobic peptide amyloid-β (Aβ), which is derived from the amyloid precursor protein (APP) by the successive action of β- and γ-secretase [1,2,3]. Due to multiple-site cleavages within APP, Aβ peptides can vary in length, with Aβ38, Aβ40 and Aβ42 (indicating 38 to 42 amino acids) representing the most abundant Aβ species [2,4]. Besides Aβ production, Aβ elimination, involving the enzymatic degradation and the transport of the peptide, is a potent determinant of cerebral Aβ levels and amyloid pathology [5,6].

Several in vitro and cell-based studies demonstrated that insulin-degrading enzyme (IDE) (EC 3.4.24.56), a zinc-requiring metalloprotease, efficiently degrades Aβ peptides [7,8,9,10]. Besides Aβ, IDE cleaves several other short polypeptides including insulin, glucagon and amylin and, thus, additionally contributes to the regulation of the carbohydrate metabolism [11,12]. IDE also represents one of the major Aβ-degrading enzymes in vivo. IDE-deficient mice show increased cerebral Aβ levels along with hyperinsulinemia and glucose intolerance, while amyloid plaque formation is decreased in the brain tissue of IDE-overexpressing animals [13,14,15]. Several studies highlighted a strong association among IDE level/-activity, Aβ accumulation and cognitive impairment in humans as well [9,16,17,18]. In line with this, some indications for a genetic association between human IDE-haplotypes and AD have been found [19,20,21,22].

Despite its predominant presence in the cytosol, IDE is localized in some organelles (endosomes, peroxisomes, mitochondria), the plasma membrane and the culture medium of microglial and neuronal cell lines [10,11,12,23,24]. The enzyme seems to be released via an unconventional pathway in association with exosomes [25,26,27] and has been detected in human serum and cerebrospinal fluid [10,28]. While IDE-dependent Aβ degradation mainly takes place extracellularly, insulin is degraded intracellularly by the enzyme after its receptor-mediated internalization [11,12]. Several cellular signals and molecules participate in the regulation of IDE gene expression and activity. These include, among others, insulin, glucagon, cellular stress signals, adenosine triphosphate (ATP) and the APP intracellular domain (AICD) [11,29]. In a pioneering work of Hamel et al., modulatory effects of several common free fatty acids (FAs) and their acyl-coenzyme A thioesters on IDE-dependent insulin degradation were described as well. This might be based on a direct interaction between FAs and a putative “cytosolic fatty-acid binding proteins signature” within the protein [30]. In line with this, we found polyunsaturated fatty acids (PUFAs) to strongly affect the enzyme, resulting in enhanced enzymatic Aβ degradation [31,32]. The latter might contribute to the beneficial effects of dietary ω3-PUFAs in preventing AD or slowing down its progression [33,34].

In general, lipids seem to play an important role in the pathogenesis of AD. There are extensive alterations in the lipid and fatty acid (FA) composition of human brain tissue affected by the disease. This might influence Aβ production, since a strong effect of the lipid environment on proteolytic APP-processing is well established [33,34]. In contrast, the impact of lipid homeostasis on Aβ-degrading mechanisms is largely unknown. Therefore, in the present study, we analyzed the effect of FA acyl chain length on Aβ degradation. This is of particular interest as medium-chain FAs (MCFAs), similarly to PUFAs, have been reported to be advantageous in mild cognitive impairment (MCI) and early stages of AD. MCFAs are saturated FAs (SFAs) consisting of 6 to 12 carbon atoms, which can be nutritionally administered as medium-chain triglycerides (MCTs) or coconut oil. The effects of MCFAs in helping ameliorate the cognitive decline caused by AD are generally attributed to the elevation of circulating ketone bodies, compensating for the impaired cerebral glucose metabolism [35,36,37,38,39,40,41,42,43]. However, we recently found that the MCFA decanoic acid (10:0) promotes neuronal health independent of ketone levels by reducing oxidative stress levels [44]. The results of the present study revealed that MCFAs might additionally act by stimulating IDE-dependent Aβ degradation, while saturated very long-chain FAs (VLCFAs) have the opposite effect.

## 2. Materials and Methods

### 2.1. Chemicals and Reagents

All chemicals and reagents were obtained from Merck/Sigma-Aldrich if not stated otherwise.

The following phospholipids were purchased from Avanti Polar Lipids (Alabaster, AL, USA): PC10:0/10:0 (1,2-didecanoyl-sn-glycero-3-phosphocholine), PC12:0/12:0 (1,2-dilauroyl-sn-glycero-3-phosphocholine), PC14:0/14:0 (1,2-dimyristoyl-sn-glycero-3-phosphocholine), PC16:0/16:0 (1,2-dipalmitoyl-sn-glycero-3-phosphocholine), PC18:0/18:0 (1,2-distearoyl-sn-glycero-3-phosphocholine), PC20:0/20:0 (1,2-diarachidoyl-sn-glycero-3-phosphocholine), PC22:0/22:0 (1,2-dibehenoyl-sn-glycero-3-phosphocholine) and PC24:0/24:0 (1,2-dilignoceroyl-sn-glycero-3-phosphocholine). These are referred to as PC10:0, PC12:0, PC14:0, etc., in the following.

Due to the limited solubility of phosphatidylcholine (PC) species containing VLCFAs, all used phospholipids were uniformly dissolved in pre-warmed (37 °C) ethanol (EtOH) to a final concentration of 2 mM by vortexing and were stored long-term in liquid nitrogen in glass bottles.

### 2.2. Cell Culture

Mock-transfected control mouse neuroblastoma Neuro2a cells (Neuro2a control) and stable IDE knock-down cells (Neuro2a IDE KD) [31,45] were cultivated in Dulbecco’s modified Eagle’s medium (DMEM high glucose) containing 10% fetal calf serum (FCS), 0.1 mM non-essential amino acid solution (MEM), penicillin/streptomycin (100 U/mL and 0.1 mg/mL), 2 mM L-glutamine, 1 mM sodium pyruvate and 400 µg/ mL hygromycin B. Cells were passed by detachment with trypsin/EDTA. Cells were checked for mycoplasma before the cell lines were transferred to the laboratory and after the performance of the experiments utilizing the PCR Mycoplasma Test Kit I/C (PromoKine, Heidelberg, Germany).

To reduce the lipid content of the cell culture medium and cell proliferation, incubation of cells with phospholipids was performed in cell culture medium with reduced FCS content (DMEM/0.1% FCS) as described earlier [31,44]. Cells used for the propidium iodide assay were treated with DMEM/0.1% FCS containing no phenolred. Cells were grown until confluency and kept in DMEM/0.1% FCS for 6 h prior to incubation. Pre-warmed (37 °C) DMEM/0.1% FCS was supplemented with phospholipids in glass vials under continuous vortexing. Then, cells were treated with DMEM/0.1% FCS containing the phospholipids in a final concentration of 10 µM for 18 h. Final EtOH concentration in the incubation medium was 5‰. In line with previous data [46], the cellular uptake measurement revealed an efficient uptake of all supplemented phospholipids, resulting in at least a threefold increase above the endogenous level (Supplementary Figure S1).

### 2.3. Murine Serum Samples

Serum samples derived from APPswe/PS1ΔE9 mice [47] whose diets were supplemented with coconut oil were provided by Heikki Tanila (Kuopio, Finland). For the feeding experiment, male, 8-week-old APPswe/PS1ΔE9-mice of C57Bl/6J background were housed in two groups. Animals were fed with a diet enriched in coconut oil or an isocaloric control diet enriched in high oleic sunflower oil over a period of 10 weeks (composition can be found in Supplementary Table S1). The environmental parameters were adjusted to a temperature at 20–22 °C, humidity at 50–60% and lights on for 12 h every day. Water and food were freely available throughout the study. At the end of the study the mice were deeply anesthetized with a pentobarbital/chloralhydrate cocktail (each 60 mg/kg, i.p.). A blood sample (~300 µL) was collected from the heart and spun in a centrifuge at 3000× *g* for 1 min to obtain the serum.

### 2.4. Collection of Conditioned Cell Culture Medium and Preparation of Cell Lysates

Conditioned cell culture medium was collected and centrifuged for 5 min at 13,000× *g* and 4 °C in order to remove cell debris. Supernatant was used for further experiments. For the detection of extracellular IDE, conditioned cell culture medium was enriched by using Amicon Ultra Filters with a cutoff of 30 kDa prior to gel electrophoresis.

After washing cells three times with ice cold phosphate buffered saline (PBS, 2.7 mM KCl, 1.8 mM KH_2_PO_4_, 137 mM NaCl, 10.1 mM Na_2_HPO_4_ × 2 H_2_O, pH 7.4), cells were scraped off in PBS and centrifuged at 13,000× *g* and 4 °C. Supernatant was removed and cells were lysed in cell lysis buffer (150 mM NaCl, 50 mM Tris/HCl pH 7.4, 2 mM EDTA, 0.1% NP-40, 0.1% Triton-X 100) containing cOmplete protease inhibitor cocktail (EDTA-free) on ice for 60 min. Samples were centrifuged again for 5 min at 13,000× *g* and 4 °C and supernatants were used for further experiments.

### 2.5. Measurement of Total Aβ Degradation

Total Aβ degradation was measured as described earlier [31,45]. Briefly, confluent Neuro2a cells were pretreated with phospholipids for 18 h as described above. Without further washing of the cells, incubation medium (DMEM/0.1% FCS + 10 µM phospholipids) was exchanged for DMEM/0.1% FCS + 10 µM phospholipids + 0.5 µg/mL synthetic human Aβ40 peptides (Botond Penke, Szeged, Hungary) (identical stock solution used for all experiments), which was incubated on cells for a further 6 h. Remaining human Aβ40 peptides in the cell culture supernatant were detected by utilizing the human Aβ40 ELISA Kit (Thermo Fisher Scientific, Schwerte, Germany) according to the manufacturer´s protocol or by Western Blot analysis using the antibody WO2 as described below. This antibody exclusively detects human Aβ peptides and thus not the endogenous murine Aβ produced by Neuro2a cells. The level of remaining human Aβ40 in the cell culture supernatant is shown in figures if not stated otherwise. Linearity of Aβ40 detection using Western Blot was verified by blotting human Aβ40 in a concentration range of 0.5 µg/mL to 0 µg/mL (0.5, 0.25, 0.125, 0.05, 0.025 and 0 µg/mL) and quantifying the band intensities.

### 2.6. Determination of Cell Viability

Viability of Neuro2a cells after incubation with phospholipids was determined by measuring both the lactate dehydrogenase (LDH) activity in the cell culture supernatant and the staining of dead cells with propidium iodide.

LDH is a cytoplasmic enzyme which is rapidly released into the extracellular compartment when the plasma membrane is damaged [48]. LDH activity in the cell culture medium of cells treated with phospholipids was determined by using the Cytotoxicity Detection KitPLUS (Roche Diagnostics, Mannheim, Germany) according to manufacturer’s instructions. A standard curve was prepared by using the cell culture supernatant of comparably confluent cells permeabilized with Triton X-100 (1%). Resulting absorbance was measured at a wavelength of 491 nm in a Safire^2^ Fluorometer (Tecan, Crailsheim, Germany).

Propidium iodide is a small fluorescent molecule that binds to DNA but cannot passively traverse into cells that possess an intact plasma membrane [49]. To assess cell viability by using propidium iodide, cells were incubated with phospholipids in DMEM no phenolred/0.1% FCS as described above. Afterwards, propidium iodide (10 µM) was added to the incubation medium and incubated with cells for 10 min at 37 °C. Resulting fluorescence was measured at an excitation wavelength of 510 ± 20 nm and an emission wavelength of 617 ± 20 nm in a Safire^2^ Fluorometer. To measure the total cell number in each well, cells were lysed by adding Triton X-100 in a final concentration of 0.5% before fluorescence measurement was repeated.

### 2.7. Quantitative Real-Time PCR

Cells were seeded on 6-well plates and treated with phospholipids as described above. Total RNA was extracted from cells by using Trizol Reagent (Thermo Fisher Scientific, Schwerte, Germany) according to manufacturer´s guidelines. The purity and concentration of the isolated RNA were analyzed by absorbance measuring using a Nano Drop 2000 (Thermo Fisher Scientific, Schwerte, Germany). Only samples with a 260 nm/280 nm and a 260 nm/230 nm ratio ≥ 1.8 were used. Reverse transcription of 2 µg RNA was carried out by utilizing the High-Capacity cDNA Reverse Transcription Kit (Thermo Fisher Scientific, Schwerte, Germany) according to manufacturer´s protocol. Quantitative real-time PCR (RT-PCR) was performed with the Fast SYBR Green Master Mix (Thermo Fisher Scientific, Schwerte, Germany) and a PikoReal Real-Time PCR System (Thermo Fisher Scientific, Schwerte, Germany). Changes in IDE gene expression (IDE) were calculated with the 2-(ΔΔCt) method [50] after normalization to β-actin gene expression (ACTB). The following primer pairs (Eurofins MWG Operon, Eberberg, Germany) were used: ACTB: 5′-CCTAGGCACCAGGGTGTGAT-3′ and 5′-TCTCCATGTCGTCCCAGTTG-3′; IDE: 5′-GCTACGTGC AGAAGGACCTC-3′ and 5′-TGGACGTATAGCCTCGTGGT-3′.

### 2.8. Measurement of Total Protein Content

Total protein concentration in samples was determined by using bicinchoninic acid (BCA) and CuSO_4_ according to Smith et al., [51]. Briefly, 20 μl of bovine serum albumin (BSA) in a concentration range of 0.1–1.2 μg/μL were used for the generation of a standard curve. For measuring samples’ protein content, 1–2 μL of each sample was pipetted in triplicate onto a 96-well plate. 200 μL of reagent buffer (CuSO_4_: bicinchoninic acid; 1:39) was added to each well. After incubation for 15 min at 37 °C and further 15 min at room temperature while shaking, absorbance was measured at a wavelength of 560 nm in a Safire^2^ Fluorometer.

### 2.9. Western Blot Analysis

For Western Blot analysis of IDE and β-actin protein levels, samples were prepared as described above, adjusted to equal protein amounts and loaded on 10–20% tris-tricine-gradient gels (Anamed Elektrophorese, Groß-Bieberau/Rodau, Germany). For measuring total Aβ degradation, the cell culture supernatant containing remaining human Aβ40 was also separated in tris-tricine-gradient gels. After transferring proteins onto nitrocellulose membranes, IDE, β-actin and human Aβ40 were detected with the primary antibodies ST1120 (1:2000), A5441 (1:5000) and W02 (1 μg/mL), all purchased from Merck, respectively. HRP-coupled antibodies W401 (anti-rabbit, 1:5000) (Promega, Mannheim, Germany) and P0260 (anti-mouse, 1:5000) (Dako, Hamburg, Germany) were utilized as secondary antibodies. Signal detection was performed with the enhanced chemiluminescence (ECL-) method (Perkin Elmer, Rodgau-Jügesheim, Germany) and densitometrical quantification of band intensity was carried out with Image Gauge software (Version 3.45) (Fujifilm). Total proteins were detected using Ponceau S staining as described in [52] before immunodetection.

### 2.10. Measurement of Constitutive Protein Secretion

Secreted alkaline phosphatase (SEAP) is a marker for the constitutive secretory pathway secreting proteins from cells regardless of external factors or signals [53]. Accordingly, for the measurement of continuous protein secretion, Neuro2a cells were transiently transfected with the vector pVectOZ-SEAP (OZ Biosciences SAS; Marseille, France) encoding for SEAP using Lipofectamine 2000 and Opti-MEM (both Thermo Fisher Scientific, Schwerte, Germany). Incubation of transfected cells with phospholipids was started 48 h after transfection. Culture medium was collected after phospholipid treatment as described above and boiled at 65 °C for 10 min. In order to measure SEAP activity in the cell culture supernatant, 100 µL of each sample was pipetted onto a 96-well plate and mixed with 100 µL of 1-Step PNPP solution (Thermo Fisher Scientific, Schwerte, Germany). Absorbance was measured at a wavelength of 405 nm using a Safire^2^ Fluorometer.

### 2.11. Measurement of IDE Activity in the Presence of Phospholipids

Determination of the direct effect of phospholipids on IDE enzyme activity was performed as described earlier [31]. Accordingly, 50 ng of recombinant human IDE (R&D Systems, Minneapolis, MN, USA) was in vitro-incubated for 15 min with 10 µM phospholipids in 200 µL IDE assay buffer (100 mM Tris-HCl pH 7.5, 50 mM NaCl, 10 μM ZnCl_2_) in glass vials under continuous shaking at 37 °C. Afterwards, 50 µL of the IDE/PC-mixture were transferred to a black 96-well-plate and mixed with the substrate Mca-RPPGFSAFK(Dnp)-OH (5 µM) (R&D Systems, Minneapolis, USA). Resulting fluorescence was detected continuously at an excitation wavelength of 320 ± 10 nm and an emission wavelength of 405 ± 10 nm in a Safire^2^ Fluorometer.

To analyze if IDE in the presence of the different phospholipids reduces Aβ40 directly, the experiment was replicated using human Aβ40 (0.5 µg/mL) instead of the fluorogenic substrate. Samples were incubated for 90 min in glass vials under continuous shaking at 37 °C and, afterwards, the amount of remaining human Aβ40 was determined using the human Aβ40 ELISA Kit (Thermo Fisher Scientific, Schwerte, Germany) according to the manufacturer´s protocol.

### 2.12. Measurement of IDE Activity in Murine Serum

IDE activity in serum samples was measured according to Liu et al., with minor modifications [28]. One µL serum was diluted in ninety-nine µL IDE-Assay buffer containing cOmplete protease inhibitor cocktail (EDTA-free), β-secretase inhibitor II (1 µM) and γ-secretase inhibitor IV (25 µM). Samples were pipetted onto a black 96-well plate before the substrate Mca-RPPGFSAFK(Dnp)-OH (10 µM) was added. Resulting fluorescence was detected as described above. IDE activity was calculated by subtracting the unspecific substrate turnover in presence of the IDE-inhibitor N-ethylmaleimide (NEM) (1 mM) and normalization to the protein content of each serum sample.

### 2.13. Mass Spectrometry Analysis

Measurement of phospholipid uptake by Neuro2a cells was performed by using a 4000 quadrupole linear-ion trap (QTrap) equipped with a Turbo Spray ion source (AB Sciex, Darmstadt, Germany) connected to a 1200 Agilent HPLC as published earlier [31,54].

### 2.14. Statistical Analysis

All quantified data presented here are based on an average of at least three independent experiments. The exact number of independently performed experiments is stated in the corresponding figure legend. Error bars represent standard deviation of the mean. Shapiro–Wilk test and Levene’s test were used for testing normal distribution and homogeneity of variance, respectively. Statistical significance was determined by two-tailed Student’s *t*-test or ANOVA in case of multiple comparisons. If data were not normally distributed or heteroscedastic, statistical significance was double validated and, in the case of non-normality, statistical significance was determined via Kruskal–Wallis one-way ANOVA followed by pairwise Wilcoxon test plus Bonferroni correction or, in the case of heteroscedasticity, via Welch-ANOVA followed by Games–Howell test. Correlation coefficients were calculated by using the Pearson method. Significance levels for *p*-values are as follows: * *p* ≤ 0.05; ** *p* ≤ 0.01 and *** *p* ≤ 0.001.

## 3. Results

### 3.1. FA Acyl Chain Length Affects IDE-Dependent Aβ Degradation

To analyze the effect of FA carbon chain length on total Aβ degradation, mouse Neuro2a cells (Neuro2a control) were treated with SFAs of increasing length with PC as a constant headgroup (PC10:0, PC12:0, PC14:0, PC16:0, PC18:0, PC20:0, PC22:0 and PC24:0). After preincubation with the different PC species (10 µM) for 18 h, cells were incubated with the phospholipids along with synthetic human Aβ40 peptides (0.5 µg/mL) for a further 6 h. Afterwards, the remaining, non-degraded human Aβ40 in the cell culture supernatant was quantified by western blot analysis. As shown in Supplementary Figure S2A, there was an 80.5 ± 1.0% reduction in synthetic human Aβ40 concentrations corresponding to 19.5 ± 1.0% remaining human Aβ40 after the 6 h incubation period. Considering the linear correlation (R^2^ = 0.9952, *p* < 0.001) between the quantified band intensities and synthetic human Aβ40 in the range of 0.5–0 µg/mL (Supplementary Figure S2B), one can assume that the obtained signals are within the linear range of detection.

**Figure 1:**
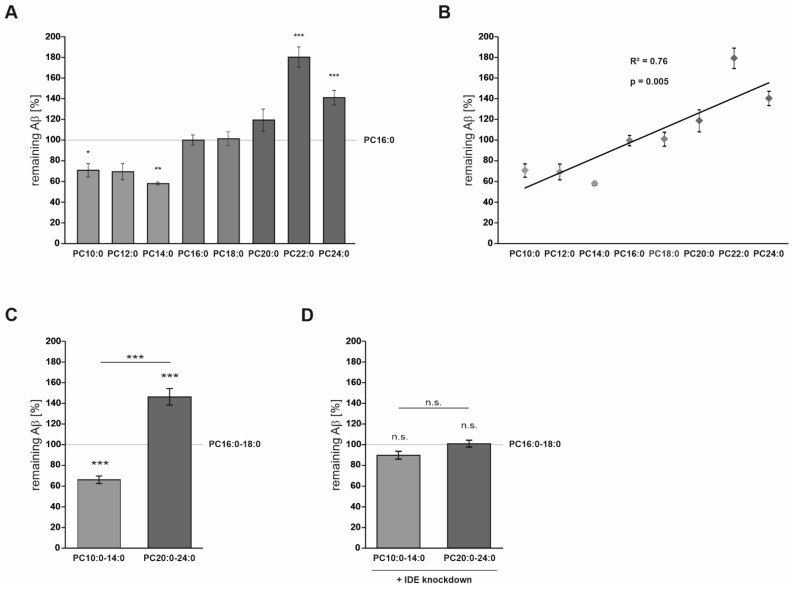
Effect of FA acyl chain length on IDE-dependent Aβ degradation. (**A**) Analysis of the effect of increasing FA carbon chain length (PC10:0, PC12:0, PC14:0, PC18:0, PC20:0, PC22:0 and PC24:0) on total Aβ degradation in mouse Neuro2a control cells compared to PC16:0 (set at 100%) (*n* ≥ 3). (**B**) Pearson correlation between the FA acyl chain length and the level of remaining human Aβ40 peptides (R^2^ = 0.76 and R = 0.87). (**C**) Pooled analysis of the effect of the examined phospholipids on Aβ degradation (PC10:0–14:0 and PC20:0–24:0 compared to PC16:0–18:0 (set at 100%), respectively) by Western Blot (*n* > 11). (**D**) Investigation of the effects of FA acyl chain length on total Aβ degradation in stably transfected Neuro2a IDE-knockdown cells (Neuro2a IDE KD) (*n* ≥ 9). Statistical significance was set as * *p* ≤ 0.05; ** *p* ≤ 0.01 and *** *p* ≤ 0.001. n.s.: not significant.

The level of remaining human Aβ40 in the medium of cells treated with the single PC species were normalized to that in the supernatant of cells treated with the control lipid PC16:0 (set at 100%). PC16:0 contains palmitic acid (16:0), one of the most abundant SFAs within the human brain [55]. Importantly, the control lipid revealed no significant effect on total Aβ degradation compared to the solvent EtOH (*p* = 0.552) (Supplementary Figure S3A). As illustrated in Figure 1A and Supplementary Figure S3B, a strong effect of FA acyl chain length on total Aβ degradation was observed. In comparison to PC16:0, PC10:0 and PC14:0 significantly reduced the level of remaining non-degraded human Aβ40 in the cell culture supernatant (*p* = 0.030 and *p* = 0.005, respectively), indicating that PC10:0 and PC14:0 increase Aβ degradation. Remaining human Aβ40 tended to be decreased in the presence of PC12:0 as well (*p* = 0.092). In contrast, the level of remaining human Aβ40 was slightly increased by PC20:0 and significantly elevated by PC22:0 and PC24:0 (*p* = 3.3 × 10^−8^ and *p* = 1.9 × 10^−4^, respectively), again, compared to PC16:0 (Figure 1A). Based on these data, a significant correlation between the FA acyl chain length and the level of remaining human Aβ40 peptides was found (R^2^ = 0.76, *p* = 0.005) (Figure 1B), and the percentage reduction of the supplemented Aβ40 peptides as a function of treatment was additionally calculated (Supplementary Figure S3C).

Since PC10:0/PC12:0/PC14:0, PC16:0/PC18:0 and PC20:0/PC22:0/24:0 had similar effects on total Aβ degradation (Figure 1A, Supplementary Figure S3B), phospholipids were pooled into three groups throughout the study: PC16:0–18:0 containing palmitic acid (16:0) and stearic acid (18:0) as the major SFAs in human brain tissue [55] (control, set as 100%), PC10:0–14:0 containing MCFAs/shorter-chained FAs and PC20:0–24:0 containing VLCFAs. Evaluation of the grouped data revealed a significant reduction of remaining human Aβ40 to 66.8 ± 3.7% in cells treated with PC10:0–14:0 compared to PC16:0–18:0 (*p* = 6.9 × 10^−6^). In contrast, PC20:0–24:0 significantly increased the level of remaining human Aβ40 peptides to 147.7 ± 8.1% in comparison to PC16:0–18:0 (*p* = 1.46 × 10^−4^); hence, the comparison of PC10:0–14:0 to PC20:0–24:0 also revealed a significant difference (*p* = 1.96 × 10^−7^) (Figure 1C). These opposite effects on Aβ40 degradation were further verified using enzyme-linked immunosorbent assay (ELISA), resulting in a significant difference in remaining Aβ40 peptides in the medium of cells treated with PC10:0–14:0 (95.3 ± 2.6%) compared to PC20:0–24:0 (105.3 ± 3.4%) (*p* = 0.035; Figure S3D). These data indicate that saturated MCFAs stimulate total Aβ degradation, while saturated VLCFAs seem to have the opposite effect. Importantly, LDH activity in the cell culture supernatant, the cellular uptake of propidium iodide and total cell numbers were found to be unaffected by the different treatments (LDH activity: *p* = 0.633; propidium iodide uptake (−Triton): *p* = 0.795; propidium iodide uptake (+Triton): *p* = 0.358) (Supplementary Figure S3E,F). Accordingly, the observed effects are not based on alterations in membrane integrity and thus cell viability caused by the different lipids.

In order to investigate whether the effects of FA acyl chain length on total Aβ degradation are dependent on IDE, the experiment was replicated by using stably transfected Neuro2a IDE-knockdown cells (Neuro2a IDE KD). IDE protein level and total Aβ degradation is strongly reduced in this cell line compared to the mock-transfected control cells (Neuro2a control) used above (Supplementary Figure S4A,B) [31,45]. As shown in Figure 1D, neither PC10:0–14:0 nor PC20:0-24:0 significantly affected total Aβ degradation in the Neuro2a IDE KD cells compared to PC16:0–18:0 (*p* = 0.129 and *p* = 0.969, respectively). In this cell line, none of the used single PC species significantly altered the total Aβ degradation in comparison to PC16:0 (Supplementary Figure S4C,D). Accordingly, and in contrast to the corresponding control cells, Neuro2a IDE KD cells displayed no significant correlation between the FA acyl chain length and the level of remaining human Aβ40 peptides (R^2^ = 0.17, *p* = 0.31) (Supplementary Figure S4E). These results indicate that saturated MCFAs/shorter-chained FAs and saturated VLCFAs affect Aβ degradation conducted by IDE.

### 3.2. FA Acyl Chain Length Affects the Secretion of IDE and Its Catalytic Activity

**Figure 2:**
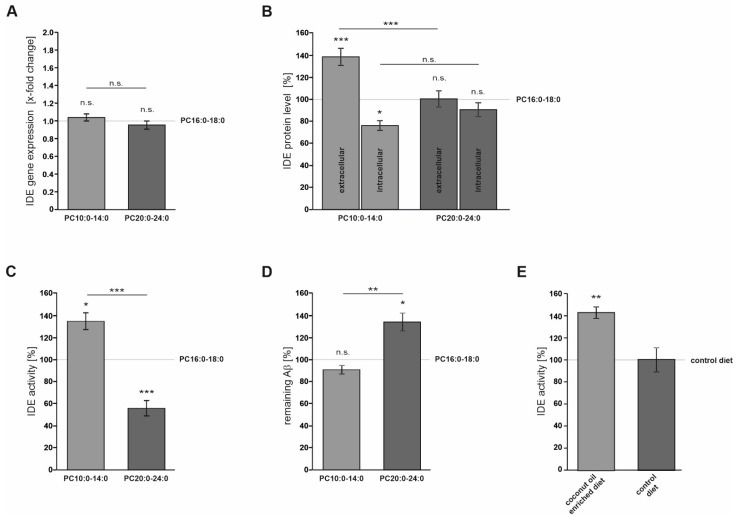
Effect of FA acyl chain length on IDE secretion and catalytic activity. (**A**) Real time PCR analysis of the effect of FA carbon chain length (PC10:0–14:0 and PC20:0–24:0 compared to PC16:0–18:0, respectively) on *Ide* gene expression in Neuro2a control cells (*n* ≥ 11). (**B**) Analysis of the impact of PC10:0–14:0 and PC20:0–24:0 on the cellular sorting of IDE in Neuro2a control cells. Extracellular and intracellular IDE protein levels were examined by Western Blot (*n* ≥ 9). (**C**,**D**) Examination of the direct effect of the FA acyl chain length on the catalytic activity of IDE by using the fluorogenic substrate Mca-RPPGFSAFK(Dnp)-OH (*n* ≥ 12) (**C**) or human Aβ40 peptides (*n* = 5) (**D**). (**E**) Effect of MCFAs on IDE activity investigated in the serum of APPswe/PS1ΔE9 mice fed with a diet enriched in coconut oil containing high amounts of MCFAs or an isocaloric control diet (*n* = 7). Statistical significance was set as * *p* ≤ 0.05, ** *p* ≤ 0.01 and *** *p* ≤ 0.001. n.s.: not significant.

MCFAs are endogenous activators of the peroxisome proliferator activated receptor γ (PPARγ). PPARγ binds to a functional peroxisome proliferator-response element (PPRE) in the IDE promoter region and has been reported to promote IDE gene transcription in primary neurons [56,57]. For these reasons, we analyzed whether IDE gene expression is affected by FA acyl chain length under the conditions chosen in the present study. RT-PCR analysis revealed that the treatment of Neuro2a control cells with both PC10:0–14:0 and PC20:0–24:0 had no impact on IDE gene expression compared to control (*p* = 0.763 and *p* = 0.717, respectively) (Figure 2A). This finding demonstrates that the FA acyl chain length-dependent effects on IDE-dependent Aβ degradation (Figure 1) are not based on alterations in IDE gene expression.

As already mentioned, IDE seems to be released into the extracellular space in association with exosomes, where it plays a major role in the catabolism of secreted Aβ peptides [24,25]. To further clarify the mechanism by which FA acyl chain length affects IDE-dependent Aβ degradation, we analyzed the impact of PC10:0–14:0 and PC20:0–24:0 on the cellular sorting of IDE. Western Blot analysis revealed that the cellular content of β-actin as control showed no significant difference for all treatments (PC10:0–14:0 vs. PC16:0–18:0: *p* = 0.716; PC20:0–24:0 vs. PC16:0–18:0: *p* = 0.952) (Supplementary Figure S5A). In contrast, a significantly elevated extracellular IDE protein level (138.9 ± 7.8%, *p* = 2.3 × 10^−4^), along with a significantly reduced intracellular IDE protein content (76.1 ± 4.4%, *p* = 0.040), were observed in cells treated with PC10:0–14:0 compared to PC16:0–18:0 (Figure 2B). Thus, the stimulated Aβ degradation in cells treated with PC10:0–14:0 might be due to an increased IDE release into the extracellular compartment. To exclude that this effect is caused by general alterations in protein secretion, SEAP activity was measured in the medium of treated cells transiently expressing SEAP. SEAP activity in the cell culture supernatant and hence protein secretion through the constitutive, unregulated secretory pathway was slightly (below 5%) altered for PC20:0–24:0 vs. PC16:0–18:0 (PC10:0–14:0 vs. PC16:0–18:0: *p* = 0.242; PC20:0–24:0 vs. PC16:0–18:0: 104.6 ± 1.49%, *p* = 0.045) (Supplementary Figure S5B). Accordingly, PC10:0–14:0 seems to specifically stimulate the release of IDE into the extracellular space resulting in an increased clearance of the supplemented human Aβ40 peptides. In contrast, PC20:0–24:0 had no effect on IDE sorting since neither the extracellular nor the intracellular IDE protein level was altered in cells incubated with PC20:0–24:0 compared to control (*p* = 1.000 and *p* = 0.571, respectively) (Figure 2B). This indicates that the reduced Aβ degradation in cells treated with PC20:0–24:0 should be attributed to another mechanism of action.

For this reason, the direct effect of the FA acyl chain length on the catalytic activity of IDE was assessed. Recombinant human IDE was in vitro-incubated with the different PC species prior to the addition of the fluorogenic substrate Mca-RPPGFSAFK(Dnp)-OH and the subsequent measurement of the resulting fluorescence. IDE activity was significantly increased to 134.9 ± 7.6% in the presence of PC10:0–14:0 in comparison to PC16:0–18:0 (*p* = 0.0133) and significantly reduced in the presence of PC20:0–24:0 (56.0 ± 6.9%, *p* = 0.0015). Thus, the comparison of PC10:0–14:0 to PC20:0–24:0 showed a significant difference as well (*p* < 0.001) (Figure 2C). The experiment was replicated utilizing synthetic human Aβ40 instead of the fluorogenic substrate, leading to similar results. ELISA measurement showed PC10:0–14:0 to slightly reduce the remaining human Aβ40 peptides compared to PC16:0–18:0 (89.6 ± 4.1%). However, this effect did not reach significance (*p* = 0.537). In contrast, PC20:0–24:0 significantly increased the non-degraded human Aβ40 compared to both PC16:0–18:0 (133.2 ± 8.2%, *p* = 0.011) and PC10:0–14:0 (*p* = 0.002) (Figure 2D). Thus, the catalytic activity of IDE seems to be directly stimulated by saturated MCFAs/shorter-chained FAs, while it is inhibited by saturated VLCFAs.

To examine whether MCFAs also affect IDE in live animals, APPswe/PS1ΔE9 mice were fed with a diet enriched with coconut oil, containing high amounts of MCFAs, or an isocaloric control diet for 10 weeks. Afterwards, IDE activity was measured in serum samples, representing the extracellular environment. As already mentioned, IDE has been detected in human serum and cerebrospinal fluid [10,28]. It is one of the major proteases involved in the cleavage of the fluorogenic substrate Mca-RPPGFSAFK (Dnp)-OH in human serum reflecting the degradation of Aβ [28]. Since this substrate is not specific to any particular protease, IDE activity was calculated by subtracting the unspecific background of the substrate turnover determined in the presence of the IDE-inhibitor NEM. As illustrated in Figure 2E, the catalytic activity of IDE was significantly increased to 142.4 ± 5.0% in the serum of mice fed with the coconut oil-enriched diet compared to control chow (*p* = 4.32 × 10^−3^). These data show that the supplementation of saturated MCFAs also increases the activity of IDE in the extracellular compartment in an ex vivo model.

## 4. Discussion

The relationship between Aβ generation and degradation determines the cerebral Aβ accumulation, which is one of the major histopathological hallmarks of AD [5,6]. While it is well established that Aβ production is strongly affected by lipids and FAs [33,34], less is known about the impact of the lipid environment on Aβ degradation. Therefore, in the present study, we analyzed the effect of FA acyl chain length on the enzymatic degradation of the peptide.

Neuro2a cells were exposed to saturated FAs with an acyl chain length ranging from 10 to 24 C-atoms before total Aβ degradation was measured. PC represents the most abundant phospholipid in mammalian cellular membranes [58], palmitic acid (16:0) and stearic acid (18:0) are the major SFAs in human brain tissue [55]. Correspondingly, FAs were applied as PCs containing identical FAs in the sn1- and sn2-position, and PC16:0 or PC16:0–18:0 (for the grouped evaluation) were chosen as controls. This experimental setup enabled us to analyze the impact of the FA acyl chain length regardless of possible effects of the choline headgroup or the glycerophosphoric acid. Liposomes containing the used phospholipids are efficiently taken up by cells. This results in the incorporation of the supplemented PCs into cellular membranes, probably affecting, e.g., membrane fluidity and structure, or their phospholipase A-dependent hydrolysis into lysolipids and free FAs [59].

**Figure 3:**
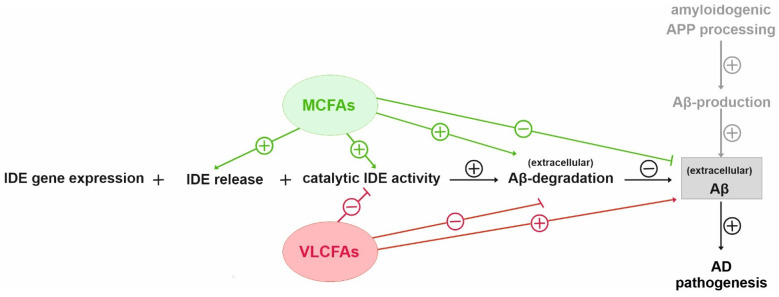
Schematic overview of the proposed effects of MCFAs and VLCFAs on IDE dependent Aβ degradation. The increased Aβ degradation in the presence of PC10:0–14:0 seemed to be based on changes in IDE sorting and a direct stimulating effect on IDE activity. In contrast, PC20:0–24:0 decreased the IDE-dependent Aβ degradation, probably by directly inhibiting the catalytic activity of the enzyme.

In Neuro2a cells, PCs containing MCFAs and shorter-chained FAs (PC10:0–14:0) significantly stimulated the degradation of exogenous human Aβ40 peptides conducted by IDE, one of the major Aβ-degrading proteases [10,13,15]. The elevated Aβ degradation in the presence of PC10:0–14:0 was accompanied by changes in IDE sorting and a direct stimulating effect on IDE activity (Figure 3). PC10:0–14:0 was found to specifically increase the release of IDE into the extracellular space, a process which occurs at least partially in association with exosomes [25,26]. IDE exosomally released by Neuro2a has been reported to be proteolytically active as the inhibition of exosome release leads to increased endogenous Aβ levels in the cell culture supernatant [25]. Considering that both exosome release in general and exosome-associated IDE secretion are known to be strongly affected by various lipids [26,31,60,61,62,63], one can assume that saturated MCFAs and shorter-chained FAs stimulate the exosomal IDE secretion into the extracellular compartment. This might lead to the elevated degradation of synthetic human Aβ40 peptides added to the medium of cells treated with PC10:0–14:0, which could be further strengthened by the increased IDE activity in presence of these PC species. In this context, a recent publication of Song et al. should be mentioned which reports that IDE is not secreted from cultured cells, but rather released nonspecifically as a consequence of reduced membrane integrity. In this study, only ~1% of total cellular IDE was released from HEK-293 and BV-2 cells. In the conditioned medium of intact Neuro2a cells, the enzyme was almost undetectable, even after concentrating the samples 10-fold [64]. In contrast, we and others [25] found measurable amounts of IDE in the cell culture supernatant of this cell line. Possible reasons for these divergent results include differences in the used growth medium, the cell to medium ratios, the methods for the concentration and detection of IDE as well as clonal heterogeneity. Further, Song et al. found LDH and two other measured cytosolic markers (glyceraldehyde dehydrogenase (GAPDH) and pitrilysin) to be released at the same relative levels as IDE from HEK-293 and BV-2 cells treated with lovastatin, implying that IDE is released nonspecifically by lysed cells [64]. However, unlike lovastatin, PC10:0–14:0 seems to specifically stimulate the secretion of IDE since it did not affect the release of SEAP, a marker for the constitutive secretory pathway [53]. Additionally, two established indicators for cytolysis, the activity of LDH in the conditioned cell culture medium and the cellular uptake of PI [48,49], were unaltered by the different phospholipids. Altogether, these data make a disruption of plasma membrane integrity very unlikely as the cause for the increased IDE release from cells treated with PC10:0–14:0. This is in line with other studies reporting a release of IDE into the cell culture supernatant in the absence of detectable LDH [10,25]. The reason for these differences is unclear but could be based on differences in the cell lines used and the cytotoxic potential of the applied stimulants.

On the other hand, the saturated VLFAs contained in PC20:0–24:0 significantly reduced the IDE-dependent Aβ degradation, probably by directly inhibiting the catalytic activity of the enzyme (Figure 3). In line with these data, an in vitro-inhibition of IDE-dependent insulin degradation by long-chain free FAs (C16–C20) with IC50-values ranging from 10 to 50 µM has been reported in the study by Hamel et al., mentioned above [30]. A direct interaction between FAs and IDE is further supported by the suspected “cytosolic fatty-acid binding proteins signature” within the enzyme and our previous observation that different FAs distinctly bind to recombinant IDE [30,31]. Altogether, our cell culture data indicate that saturated MCFAs and shorter-chained FAs are more beneficial than saturated longer-chained FAs and VLCFAs with respect to IDE-dependent Aβ degradation.

An increased IDE activity was also observed in the serum of mice upon dietary supplementation of MCFAs in the form of coconut oil. We would like to point out that the effects obtained with transgenic mice fed with coconut oil are comparable to the effects observed in cell culture. By utilizing an IDE knock-down, we could confirm that the major effect of coconut oil is linked to the Aβ degrading enzyme IDE. Due to limitation of samples size in mice, this confirmation which would be ideally done by a specific inhibitor of IDE (e.g., 6bK) was not possible. Therefore, we cannot rule out that, in vivo, other proteases besides IDE are affected by coconut oil, which is a potential caveat in our study. In addition, we suggest further studies investigating whether mutations in the “cytosolic fatty acid binding protein signature” would reinforce the notion that MCFAs regulate IDE. In order to address this question, further research is warranted.

IDE activity in murine serum samples was determined by using the fluorogenic substrate Mca-RPPGFSAFK (Dnp)-OH (also called substrate V) and the subsequent subtraction of its turnover in the presence of the IDE inhibitor NEM. In this context, it should be noted that both the used substrate and NEM are not specific for IDE: substrate V can be cleaved by several other metalloproteases as well and NEM acts as a general irreversible inhibitor of cysteine peptidases. However, IDE and angiotensin converting enzyme (ACE) are the major proteases in human serum to mediate substrate V degradation, as reported by Liu et al. Among several inhibitors tested, only insulin, amylin, EDTA and the ACE inhibitor lisinopril had the ability to inhibit substrate V degradation in human serum. The existence of IDE activity in these samples was further confirmed by immunocapture of IDE using monoclonal antibodies. Importantly, in the same study, substrate V turnover in serum was strongly inhibited by increasing doses of Aβ and thus seems to reflect Aβ degradation [28]. Altogether, these data indicate that IDE is one of the major serum proteases affected by MCFAs/coconut oil. Nevertheless, we cannot completely rule out the possibility that ACE activity is also altered by the coconut oil-enriched diet. Utilizing short fluorogenic peptides as substrates is discussed as having further disadvantages [65]. In particular, under certain conditions, using small fluorogenic peptides to monitor IDE activity might result in the identification of “false-positive” or “false-negative” IDE modulators. Therefore, an independent confirmation of the identified IDE activity modulators is extremely important. In this study, we confirmed our results by using an IDE knock-down cell line. Effects observed in the presence of MCFAs were attenuated or disappeared in the absence of IDE, suggesting that IDE is the major protease responsible for the MCFA-affected Aβ degradation. In detail, to analyze Aβ degradation with a chemically not-modified substrate, we used N2a cells. N2a cells are mouse neuroblastoma cells producing Aβ peptides that are not or are in very low amounts detected by the antibody WO2, specific for human Aβ. These mouse cell lines were incubated with human Aβ peptides in the presence or absence of MCFAs. WB analysis using the WO2 antibody revealed that the remaining human Aβ in the presence of MCFAs is decreased, further suggesting that MCFAs increase Aβ degradation in an assay which is not influenced by fluorogenic substrates. Notably, this effect could be not observed in IDE knock-down N2a cells.

Considering the observed effects of MCFAs on IDE in Neuro2a cells, the increased serum IDE activity upon dietary coconut oil supplementation might be based on an enhanced IDE release into the extracellular space in combination with a direct stimulation of its catalytic activity. The increased serum IDE activity after MCFA supplementation probably leads to reduced peripheral Aβ levels. According to the ‘peripheral sink hypothesis,’ the latter should be associated with an increased Aβ efflux from brain tissue [66,67]. In line with this and further emphasizing the relevance of our results, Liu et al. reported a negative association between probable AD and MCI and the activity of serum proteases mediating Aβ degradation measured by using the same substrate [28]. As MCFAs are able to cross the blood brain barrier [68,69], one might speculate that they could also affect the secretion and activity of IDE in the central nervous system. This would be of particular importance since extracellular IDE might play a key role in the clearance of extracellularly accumulating Aβ peptides within brain tissue, which are closely linked to AD pathology. It should be additionally mentioned that a trend towards decreased total Aβ levels in the parietal lobe of dogs, dietarily administrated with MCTs, has been reported. However, in this study, Aβ degradation was not analyzed and the tendentially reduced cerebral Aβ levels after MCT-supplementation were attributed to a lessened APP protein level [70]. Beside Aβ degradation mediated by IDE, hepatic clearance of Aβ peptide might also be an important factor and may be influenced by coconut oil, which should be addressed in further studies. In detail, on the one hand insulin can be considered as a potential competitive inhibitor for Aβ degradation as it is another substrate for IDE [71]. Therefore, reduction in insulin might result in an increased Aβ degradation by IDE. On the other hand, insulin affects LRP mediated hepatic clearance of Aβ [72], further underlying the complexicity of Aβ catabolism.

Further studies are needed to analyze the possible link between the observed increased serum IDE activity after MCFA supplementation and cerebral Aβ levels as well as the mechanism by which MCFAs might affect IDE in vivo.

In summary, the data of the present study further underline the protective properties of saturated MCFAs in respect to AD pathogenesis. Our results indicate that the beneficial effects of MCFAs for MCI- and AD-patients might not only be based on an elevation of circulating ketone bodies improving cerebral energy metabolism and the reduction in oxidative stress levels [35,36,37,38,39,40,41,42,43,44], but also by a stimulation of IDE-dependent Aβ degradation. Interestingly, an association between variants of the polymorphisms in the IDE gene and the cognitive response of AD-patients to MCT-supplementation has been described [73], further emphasizing a connection between MCFAs and IDE in humans. Considering the various substrates of IDE, an MCFA-induced elevation of IDE-activity might also affect other important pathways including those regulating carbohydrate metabolism. However, further particular clinical studies are needed to evaluate the potential of MCFAs for nutritional and pharmacological interventions regarding AD.

## Data Availability

Not applicable.

## Supplementary Materials

The following are available online at https://www.mdpi.com/article/10.3390/cells10112941/s1. Figure S1: Phospholipid uptake by Neuro2a cells, Figure S2: Western Blot analysis of total Aβ degradation, Figure S3: Effect of FA acyl chain length on cell viability and Aβ degradation in Neuro2a cells, Figure S4: Effect of FA acyl chain length on Aβ degradation in stably transfected Neuro2a IDE-knockdown cells (Neuro2a IDE KD), Figure S5: Effect of FA acyl chain length on β-actin protein level in Neuro2a cells and constitutive protein secretion, Table S1. Composition of the diet enriched in coconut oil purchased from Merck/Sigma-Aldrich.
