# Supplementary material for: Medium-Chain Length Fatty Acids Enhance Aβ Degradation by Affecting Insulin-Degrading Enzyme
Source: Cells. 2021 Oct 29;10(11):2941. doi: 10.3390/cells10112941 (PMC8616162; doi:10.3390/cells10112941)
Supplement: Supplementary file 1 [file cells-10-02941-s001.zip › cells-1292347-supplementary.pdf]

Supplementary Materials

Medium-chain length fatty acids enhance Aβ degradation by affecting insulin-degrading enzyme

Janine Mett [1], Anna A. Lauer [2], Daniel Janitschke [2], Lea V. Griebsch [2], Elena L. Theiss [2], Heike S. Grimm [2], Hennariikka Koivisto [3], Heikki Tanila [3], Tobias Hartmann [2,4] and Marcus O. W. Grimm [2,4,5*]

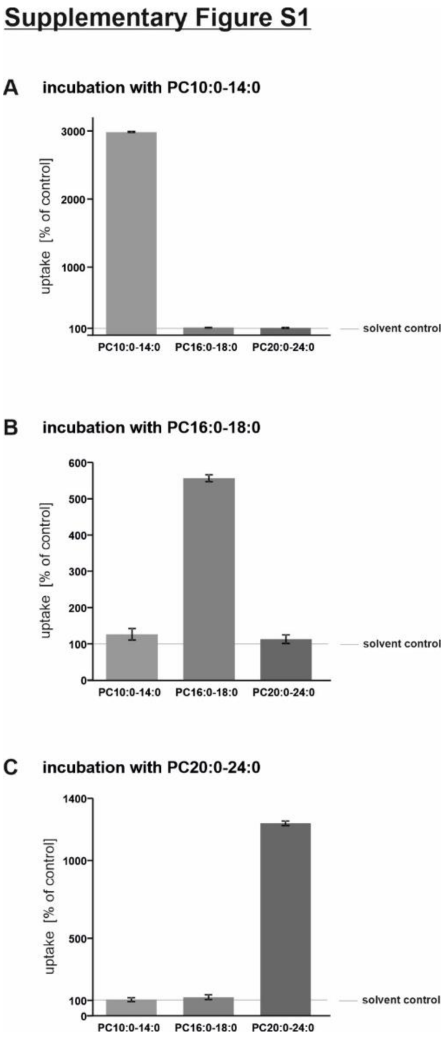

Supplementary Figure S1. Phospholipid uptake by Neuro2a cells. PC profile of Neuro2a control cells incubated with PC10:0-14:0 **(a)** (n = 3), PC16:0-18:0 (n = 3) **(b)** or PC20:0-24:0 (n = 3) **(c)** measured by mass spectrometry. No significant correlation was found between the uptake of the single PC species and the observed effects on Aβ degradation ($p = 0.541$).

Supplementary Figure S2

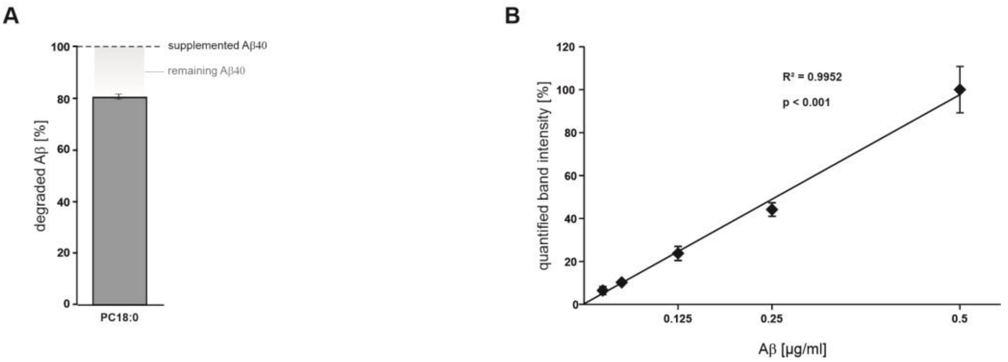

Supplementary Figure S2. Western Blot analysis of total Aβ degradation. (a) Percentage reduction of the supplemented human Aβ40 after 6 h incubation with mouse Neuro2a control cells treated with PC18:0 (n = 12). **(b)** Significant linear correlation ($R^2 = 0.9952$, $p < 0.000$) between the quantified band intensities and synthetic human Aβ40 in the range of 0.5 -0 µg/ ml (n = 4).

Supplementary Figure S3

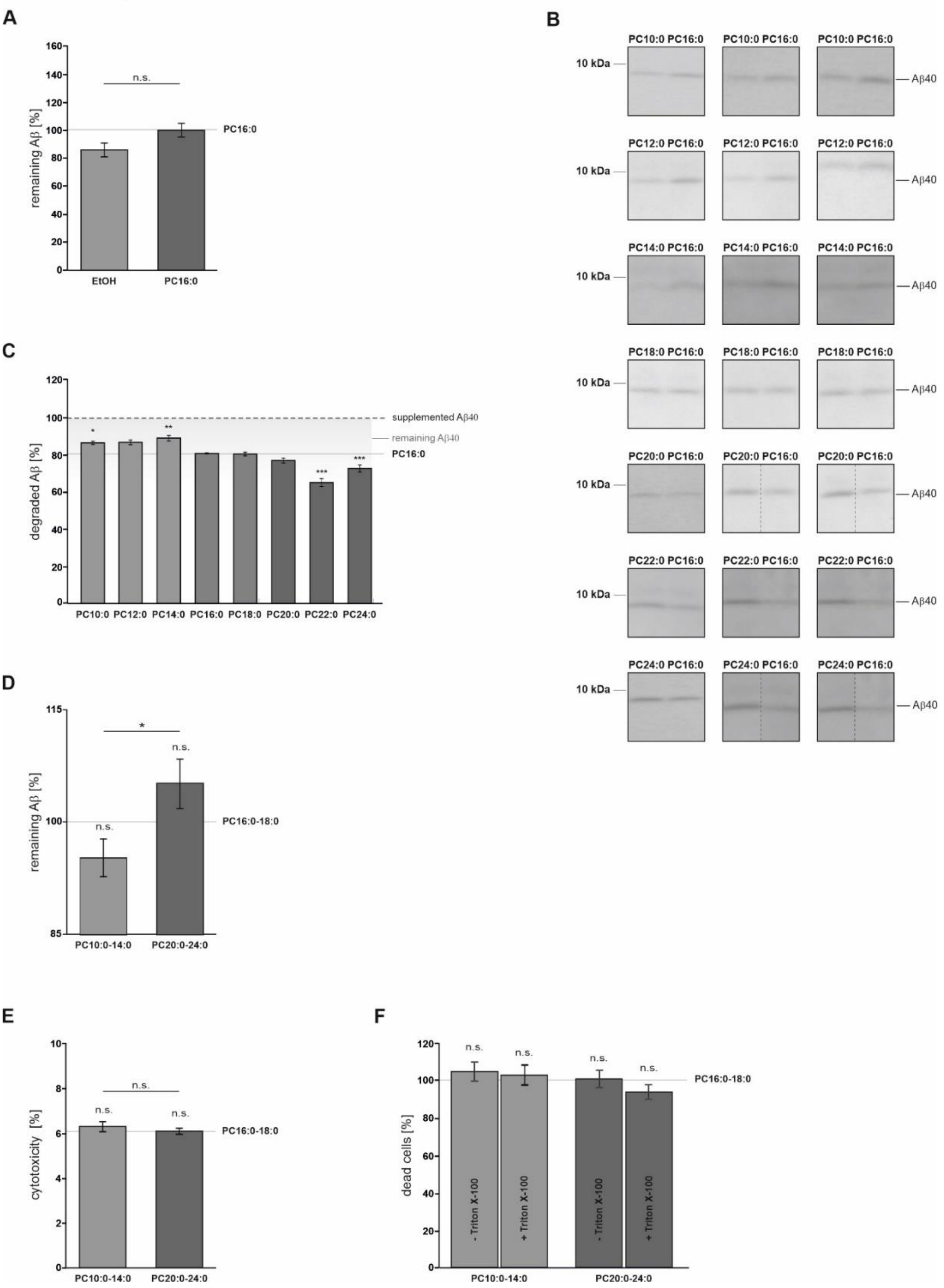

Supplementary Figure S3. Effect of FA acyl chain length on cell viability and Aβ degradation in Neuro2a cells. (a) Effect of the solvent EtOH on total Aβ degradation in mouse Neuro2a control cells compared to PC16:0 (set at 100 %) (n ≥ 6). **(b)** Representative Western blots for the effect of FA acyl chain length on Aβ degradation in Neuro2a control cells shown in Fig. 1A (n ≥ 3). **(c)** Percentage reduction in synthetic Aβ40 concentrations calculated based on the data shown in Fig. 1A (n ≥ 3). **(d)** Effect of the examined phospholipids on Aβ degradation in Neuro2a control cells measured by ELISA (n = 9). **(e)** Cytotoxicity after incubation with the examined phospholipids as indicated by LDH activity in the cell culture medium (n ≥ 9) **(f)** and cellular uptake of propidium iodide (n ≥ 25) (-Triton X-100: dead cells after PC-treatment, + Triton X-100: total cell numbers after PC-treatment). Statistical significance was set as * $p \leq 0.05$, ** $p \leq 0.01$ and *** $p \leq 0.001$. n.s.: not significant.

Supplementary Figure S4

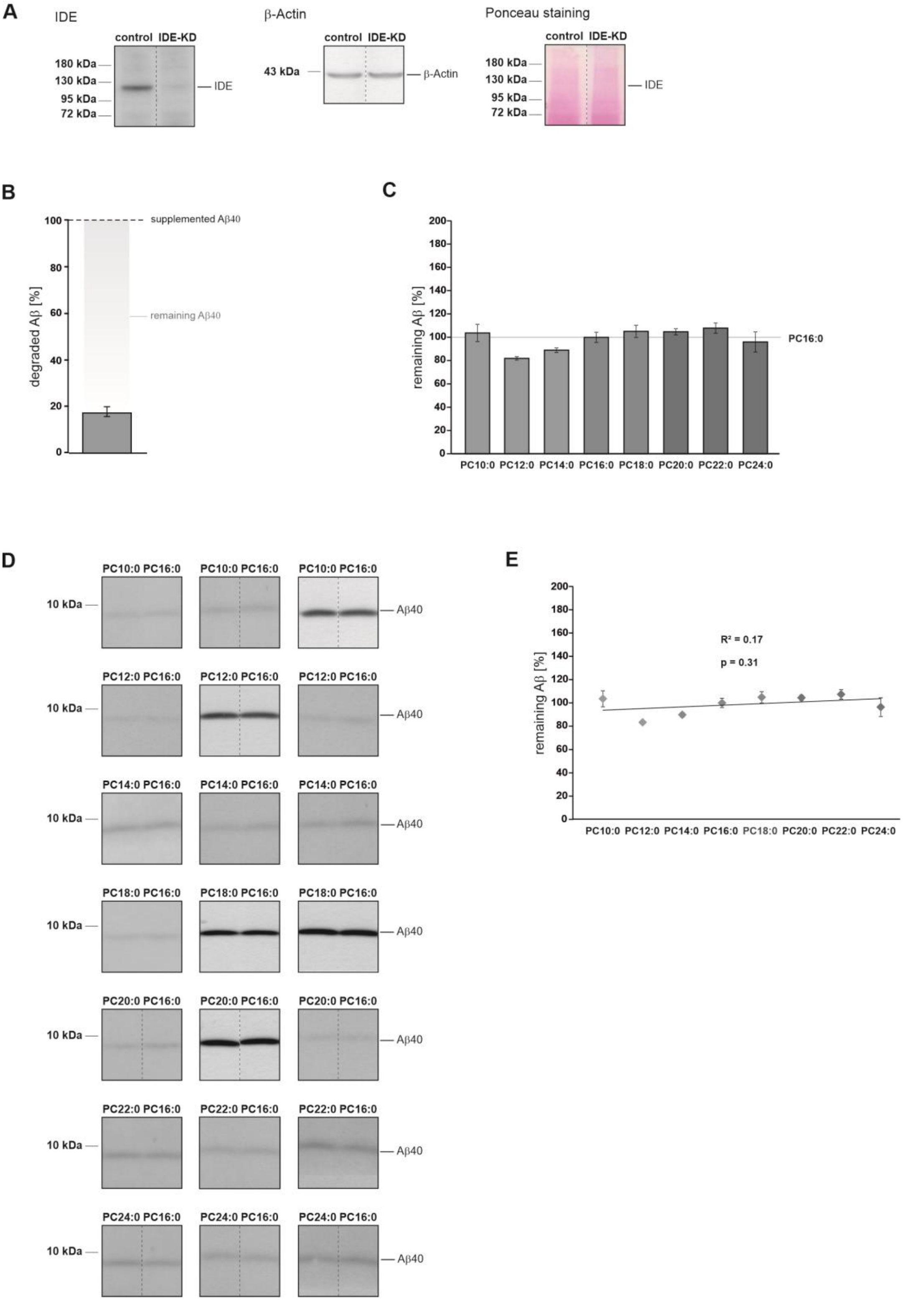

Supplementary Figure S4. Effect of FA acyl chain length on Aβ degradation in stably transfected Neuro2a IDE-knockdown cells (Neuro2a IDE KD). (a) IDE protein level in Neuro2a control and Neuro2a IDE KD cells. β-actin protein level and total protein staining with Ponceau S were used as loading controls. **(b)** Percentage reduction of the supplemented human Aβ40 after 6 h incubation with Neuro2a IDE KD (n = 5). **(c)** Effect of FA carbon chain length on total Aβ degradation in Neuro2a IDE KD cells compared to PC16:0 (set at 100 %) (n ≥ 3). **(d)** Representative Western blots for the effect of FA acyl chain length on Aβ degradation in Neuro2a IDE KD cells shown in (c) (n ≥ 3). **(e)** Correlation between the FA acyl chain length and the level of remaining human Aβ40 peptides after incubation with Neuro2a IDE KD.

Supplementary Figure S5

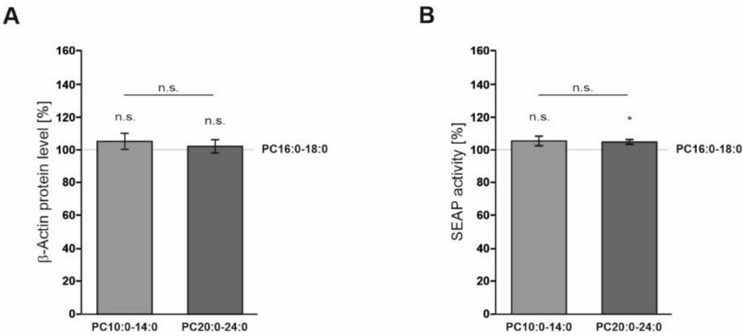

Supplementary Figure S5. Effect of FA acyl chain length on β-actin protein level in Neuro2a cells and constitutive protein secretion (a) β-actin protein level in Neuro2a control cells treated with the examined phospholipids ($n \geq 6$). **(b)** SEAP activity in the cell culture supernatant of Neuro2a control cells after incubation with phospholipids ($n = 9$). Statistical significance was set as * $p \leq 0.05$. n.s.: not significant.

Supplementary Table S1

name	FA composition
coconut oil diet	enriched in MCFAs:<br>45.4% lauric acid (12:0)<br>18.0% myristic acid (14:0)<br>10.5% palmitic acid (16:0)<br>7.5% oleic acid (18:1)<br>5.4% caprylic acid (8:0)<br>8.4% capric acid (10:0)

Supplementary Table S1. Composition of the diet enriched in coconut oil purchased from Merck / Sigma-Aldrich. Data were obtained from the CRC Handbook of Chemistry & hysics, 74th Ed. (1993-94), p. 7-29.
